# Supplementary material for: Joint changes in RNA, RNA polymerase II, and promoter activity through the cell cycle identify non-coding RNAs involved in proliferation
Source: Sci Rep. 2021 Sep 23;11:18952. doi: 10.1038/s41598-021-97909-w (PMC8460802; doi:10.1038/s41598-021-97909-w)
Supplement: Supplementary file 1 — Supplementary Information 1. [file 41598_2021_97909_MOESM1_ESM.pdf]

Supplementary methods

Supplementary method 1- RNA-seq library preparation

Briefly, 900 ng total RNA was used as starting material. The first step involved the removal of ribosomal RNA (rRNA) using biotinylated, target-specific oligos combined with Ribo-Zero rRNA removal beads. Following purification, RNA was fragmented into small pieces using divalent cations at 94°C for 4 min. First and second strand cDNAs were synthesized using random oligonucleotides and SuperScript II, followed by DNA polymerase I and RNase H. Exonuclease/polymerase was used to produce blunted overhangs. Illumina SR adapter oligonucleotides were ligated to the cDNA after 3' end adenylation. DNA fragments were enriched by 15 cycles of PCR. The libraries were purified using the AMPure XP (Beckman Coulter, Inc., Indianapolis, IN, USA), quantitated by qPCR using KAPA Library Quantification Kit (Kapa Biosystems, Inc., Wilmington, MA, USA), and validated using Agilent High Sensitivity DNA Kit on a Bioanalyzer (Agilent Technologies, Santa Clara, CA, USA). The size range of the DNA fragments was measured to be in the range of 210-850 bp and peaked around 310 bp. Prior to sequencing, the libraries were quantified (KAPA Library Quantification Kit (Illumina/ABI Prism), normalized, pooled, and size selected (Ampure XP beads). Then each pool was quantified again. The pooled purified libraries were normalized and 20 pM was subject to clustering (by a cBot Cluster Generation System on a HiSeq2500 Rapid run mode flow cell (Illumina)), according to manufacturer's instructions.

Supplementary method 2-Chromatin Immunoprecipitation (ChIP)

For each time point in the cell cycle, ~ 3.5 million HaCaT cells were washed twice with PBS, cross-linked with 0.5% formaldehyde at room temperature for 10 min, following addition of 125 mM glycine to stop the cross-linking. Cells were washed again with PBS and scraped from the plates in PBS supplemented with 1mM EDTA and protease inhibitors. Cross-linked cells were collected by centrifugation and resuspended in 1 ml cold RIPA buffer (10 mM Tris pH 8.0, 1 mM EDTA, 140 mM NaCl, 1% Triton X-100, 0.1% SDS, 0.1% Na-Deoxycholate) supplemented with protease inhibitors and 125 mM glycine. Samples were frozen in liquid nitrogen and stored at -80°C. Later, cells were thawed and sonicated, using a model 250 Sonifier (Branson Ultrasonic Corporation), at 20% duty cycle, 2.5 output control, 1 min x 10 with 30 sec pauses in between. To further digest the DNA, cells were treated with 400 U of Micrococcal Nuclease (NEB, M02475) at 37°C for 15 min. Debris was removed by centrifuging (16.000 x g) at 4°C for 15 min and soluble chromatin supernatant equivalent to 3.5×10^6 cells/IP was pre-cleared using 20 µl of a 1:1 mix of protein A and protein G Dynabeads (Invitrogen). Overnight immunoprecipitation was performed using either two µg of anti-H3K4me3 (Diagenode, C1541003-50), anti-H3K27me3 (Diagenode, C15410195), anti- Pol II (Diagenode, C15200004), or the non-specific immunoglobulin G (IgG) antibodies Mouse IgG (Diagenode, C15400001) and Rabbit IgG (Diagenode, C15410206). Antibody-protein complexes were immunoprecipitated with protein A/G Dynabeads for 3 hours at 4°C, then washed five times with RIPA buffer supplemented with protease inhibitors, once with LiCl wash buffer (250 mM LiCl, 10 mM Tris pH 8.0, 1 mM EDTA, 0.5% NP-40, 0.5% Na-Deoxycholate) supplemented with protease inhibitors, and once in TE (10 mM Tris-HCl pH 8.0, 1 mM EDTA). Immunoprecipitated DNA was reverse cross-linked by adding 1 µl RNaseA [10 mg/ml] and incubated at 37°C for 30 min. Then we added 2.5 µl 20% SDS and 5 µl Proteinase K [10mg/ml] and incubated at 55°C for 1 hour, followed by purification using QIAquick PCR purification kit (Qiagen, 28104).

Supplementary method 3- ChIP-seq library preparation

ChIP-seq libraries were prepared using the MicroPlex Library Preparation Kit v4 (Diagenode) in accordance with the manufacturer's protocol. In brief, ~1 ng immunoprecipitated dsDNA was used as input in the template preparation process that provides efficient end repair of the fragmented dsDNA input. Further, library synthesis was performed by ligation of MicroPlex patented stem-loop adapters. The stem-loop adaptors with blocked 5' ends were ligated to the 5' end of the genomic DNA, leaving a nick at the 3'end. Next, a library amplification step was performed, which enabled extension of the template, cleavage of the stem-loop adaptors, and amplification of the library. In the final step, the 3' ends of the genomic DNA were extended to complete library synthesis and Illumina-compatible indexes were added through a high-fidelity amplification. Prior to sequencing, the libraries were quantified using the KAPA Library Quantification Kit (Illumina/ABI Prism), normalized, pooled, and size selected (Ampure XP beads). Then each pool was quantified again. The pooled purified libraries were normalized, and 20 pM was subject to clustering (by a cBot Cluster Generation System on a HiSeq2500 HO flow cell (Illumina)), according to manufacturer's instructions.

Supplementary method 4- Western blot analysis

In the cell synchronization experiments, cells were washed once with cold PBS before pelleted, snap-frozen, and stored at -80°C. Total protein extracts for Pol ll analysis were prepared by resuspending cell pellets in 1 x pellet-cell-volume (PCV) of Buffer I (10 mM Tris-HCl pH 8.0, 200 mM KCl, 1 mM DTT, protease inhibitor) and 1 x PCV of Buffer II (10 mM Tris-HCl pH 8.0, 200 mM KCl, 2 mM EDTA, 40% Glycerol, 0.5% Triton X-100, 0.5% NP-40, 1 mM DTT, protease inhibitor). The mixture was gently shaken for 2 hours at 4°C, extracts were cleared by centrifugation, and protein concentrations were measured using the Bradford method (Bio-Rad). Total protein extracts for H3K4me3 and H3K27me3 analysis were isolated using Allprep DNA/RNA/protein mini Kit (Qiagen, 80004) according to the manufacturer's protocol, except that the lysis buffer in the last step was replaced with SDS buffer. The protein concentration was measured using Direct Detect assay free cards (Merc, DDAC00010-GR) and analyzed on a Direct Detect Infrared Spectrometer. Proteins were separated by electrophoresis on NuPAGE 4-12% Bis-Tris gels (Invitrogen, NP0322BOX) and blotted onto PVDF membranes by standard procedures. PVDF membrane for high molecular weight proteins (Amersham Hybond, 10600023) was used for RNA Pol II detection and PVDF membrane for low molecular weight proteins (Amersham Hybond, 10600061) was used for H3K4me3 and H3K27me3 detection. Primary antibodies for RNA Pol II (Diagenode, C15200004), H3K4me3 (Diagenode, C15410003), and H3K27me3 (Diagenode, C15410195) were diluted [1:1000] in 5% dry milk in PBS containing 0.1% Tween and membranes were incubated overnight at 4°C. Anti-beta Actin (Abcam, ab8226) [1:5000] was used as loading control. The secondary antibodies IRDye 800CW goat anti-rabbit (LI-COR Biosciences, 925-32211) and 680RD goat anti-mouse (LI-COR Biosciences, 926-68070) were used in a [1:15000] dilution and membranes were visualized using the LI-COR Biosciences detection system.

Supplementary figures and tables

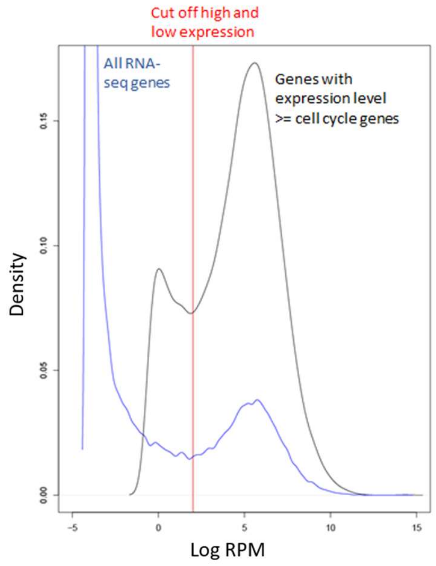

Supplementary Figure S1: Density plot of the bimodal distribution of RNA-seq expression. The blue line represents the mean expression for all genes in the RNA-seq data set (n = 65988). The black line represents genes with mean expression ≥ the cell cycle gene with lowest mean expression (n = 14065). This set of genes included cell cycle genes (n = 1803) and genes with no significant cell cycle-dependent expression pattern (!CC genes, n = 12262). The set of genes had a bimodal distribution of expression levels, where the red line represents the cut off between high and low RNA-seq expression. We divided the !CC genes into genes with high expression level (!CC_high, n = 9259) and genes with low expression level (!CC_low, n = 3003).

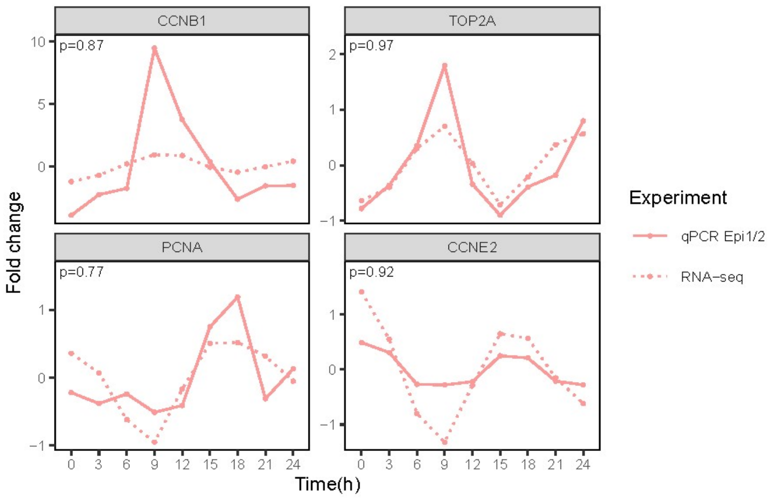

Supplementary Figure S2: Technical validation of RNA-seq expression for *CCNB1*, *CCNE2*, *PCNA*, and *TOP2A*. Spearman's correlation coefficients (ρ) were calculated based on the mean RNA-seq expression per time point and mean RT-qPCR fold change per time point from the original experiments (Epi1, Epi2).

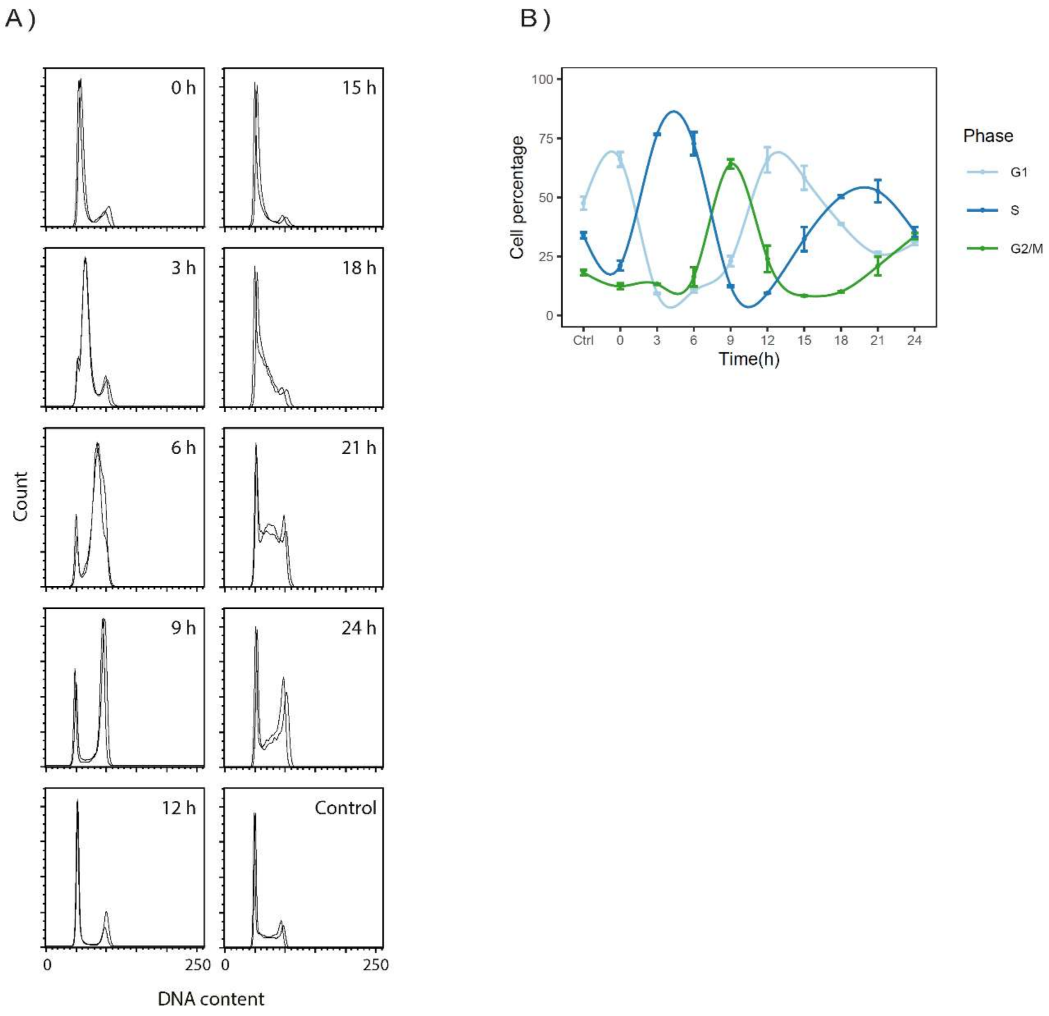

Supplementary Figure S3: Synchrony of double thymidine blocked HaCaT cells from two validation experiments (Val1, Val2). (A) Cell synchrony was monitored by flow cytometry of propidium iodide-stained cells. The figure shows superimposed DNA content profiles of the two replicate experiments for each time point. Horizontal axes show DNA content (arbitrary units) and vertical axes show the number of cells with the corresponding DNA content. Control is unsynchronized cells. (B) Percentage of cells assigned to G1, S, and G2/M phases for each of the time points analyzed. Values and error bars are averages and standard deviations (n = 2).

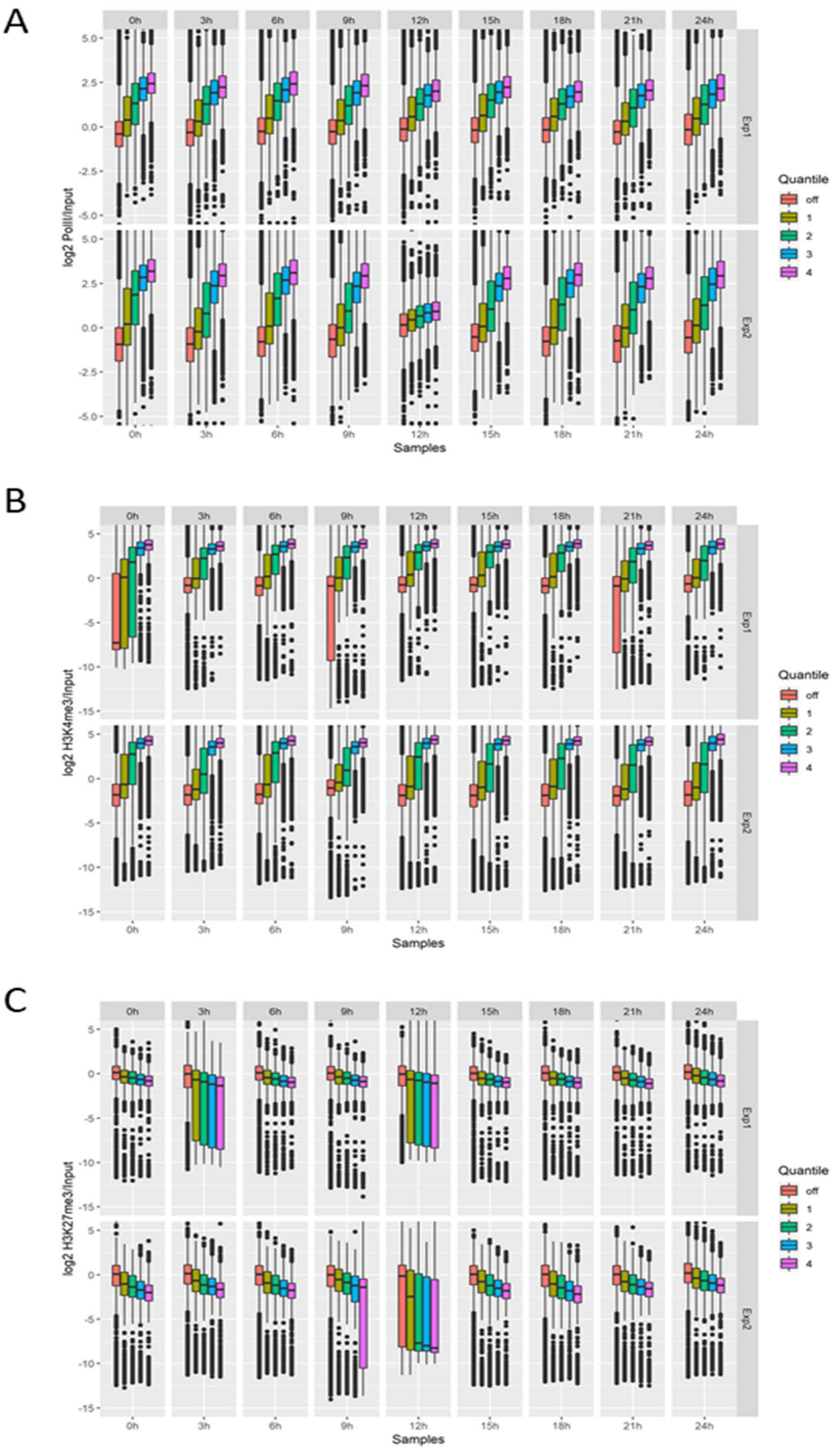

Supplementary Figure S4: Average abundance of ChIP-seq signal in promoter regions around the transcription start site (TSS). The sum of counts within each bin of size 50 around the TSS area of all annotated genes. We divided the genes into quantiles based on RNA-seq expression; off = genes not expressed, 1 = 0-25%, 2 = 25-50%, 3 = 50-75%, 4 = 75-100%. (A) Pol II ChIP-seq, (B) H3K4me3 ChIP-seq, and (C) H3K27me3 ChIP-seq.

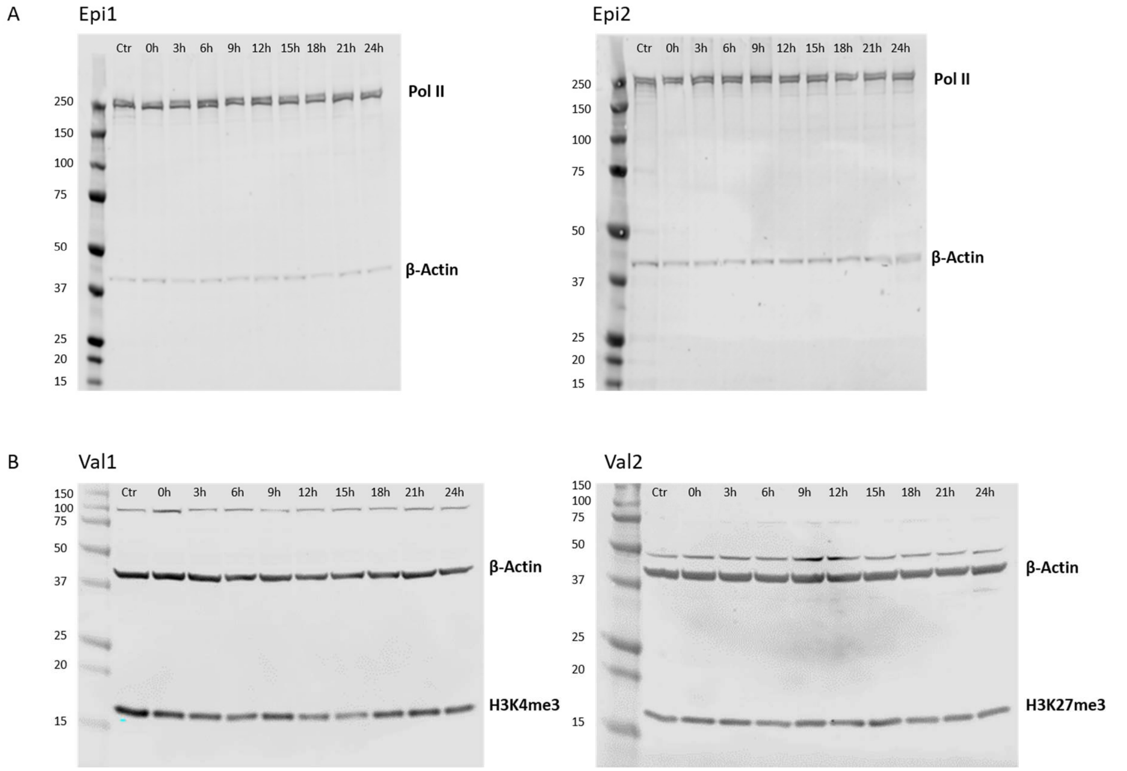

Supplementary Figure S5: Western blot analysis. (A) Pol II: Protein was isolated from the original experiments (Epi1,Epi2). (B) H3K4me3 and H3K27me3: Protein was isolated from the two validation experiments (Val1, Val2). Odyssey® Protein Molecular Weight Marker (cat.no 928-40000) was used as a ladder (10-250 kDa). Quantification (data not shown) indicated no changes during cell cycle or between the different synchronization experiments for Pol II, H3K4me3, or H3K27me3.

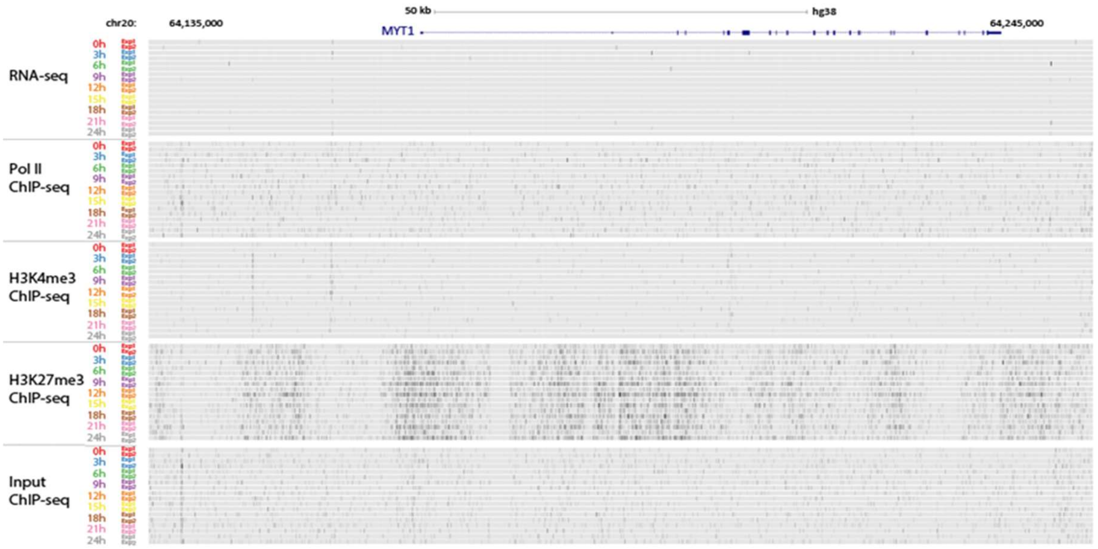

Supplementary Figure S6: The UCSC Genome browser (GRCh38/hg38) view of RNA-seq and Pol II, H3K4me3, H3K27me3, and Input ChIP-seq data at the *MYT1* (NM_004535) gene locus.

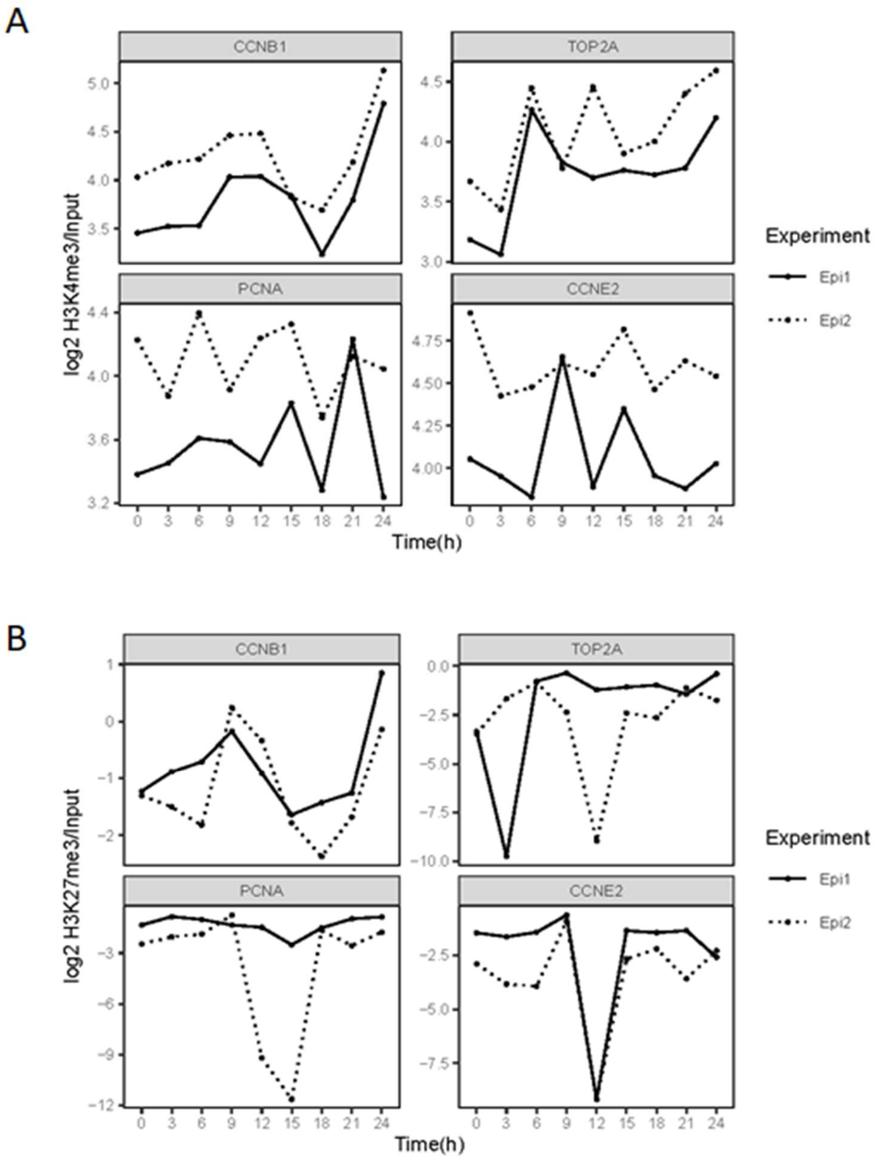

Supplementary Figure S7: (A) H3K4me3 and (B) H3K27me3 ChIP-seq profiles for the cell cycle genes *CCNB1*, *CCNE2*, *PCNA*, and *TOP2A*. Negative values on the y axis for H3K27me3 ChIP-seq indicate the ChIP-seq signal is lower than the background (input) signal.

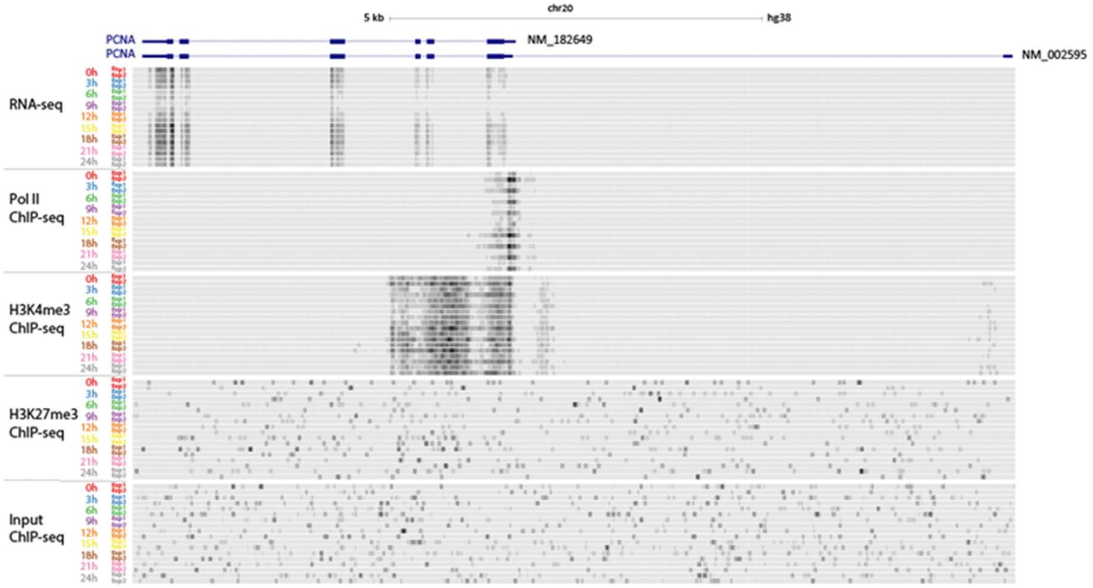

Supplementary Figure S8: The UCSC Genome browser (GRCh38/hg38) view of RNA-seq and Pol II, H3K4me3, H3K27me3, and Input ChIP-seq data at the *PCNA* (NM_182649 and NM_002592) gene locus. The Pol II and H3K4me3 data indicate that the short isoform (NM_182649) and not the long isoform (NM_002592) is being transcribed.

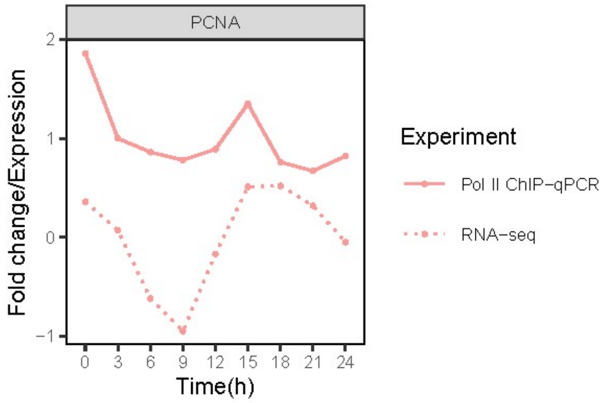

Supplementary Figure S9: *PCNA* Pol II ChIP-qPCR and RNA-seq expression. Expression is the mean RNA-seq expression per time point (Epi1, Epi2) and mean Pol II ChIP-qPCR fold change per time point from the new biological replicates (Val1, Val2).

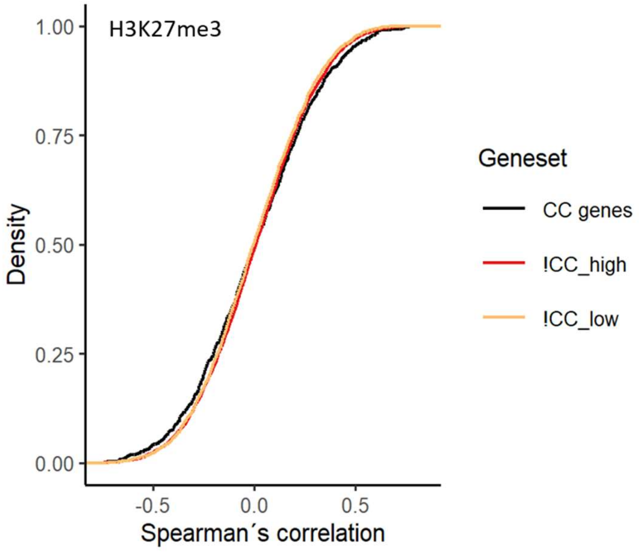

Supplementary Figure S10: Spearman's correlation analysis of RNA-seq expression against H3K27me3 ChIP-seq signal. There was no clear shift in the mean correlations for three gene sets CC genes, !CC_high, and !CC_low (*p*-values of 0.63, 0.023, and 0.84, respectively.) There were no significant differences between the gene sets (CC genes against !CC_high *p*-value = 0.72 and CC genes against !CC_low *p*-value = 0.60). Significant differences were determined by Student's *t*-test (unpaired, two-tailed) assuming unequal variances.

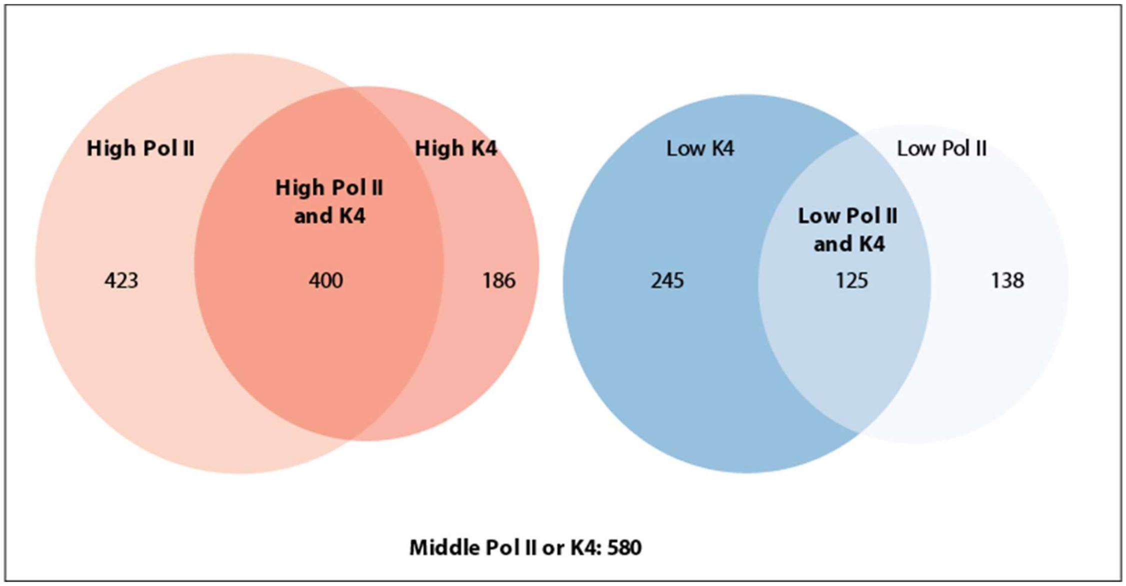

Supplementary Figure S11: Venn diagram for cell cycle genes with combined Pol II and H3K4me3 (K4) signal (n = 1714). Genes were divided in high ($\rho > 0.2$), low ($\rho < -0.2$), and middle ($\rho > -0.2$ and $\rho < 0.2$) correlated genes, based on the genes´ Spearman's correlation value for RNA-seq expression against ChIP-seq signal. We did GO analysis for five different groups (in bold); high correlation for Pol II and K4 (n = 400), high correlation for Pol II (n = 423), high correlation for K4 (n = 186), middle correlation for Pol II or K4 (n = 580), and low correlation for Pol II and K4 (n = 125).

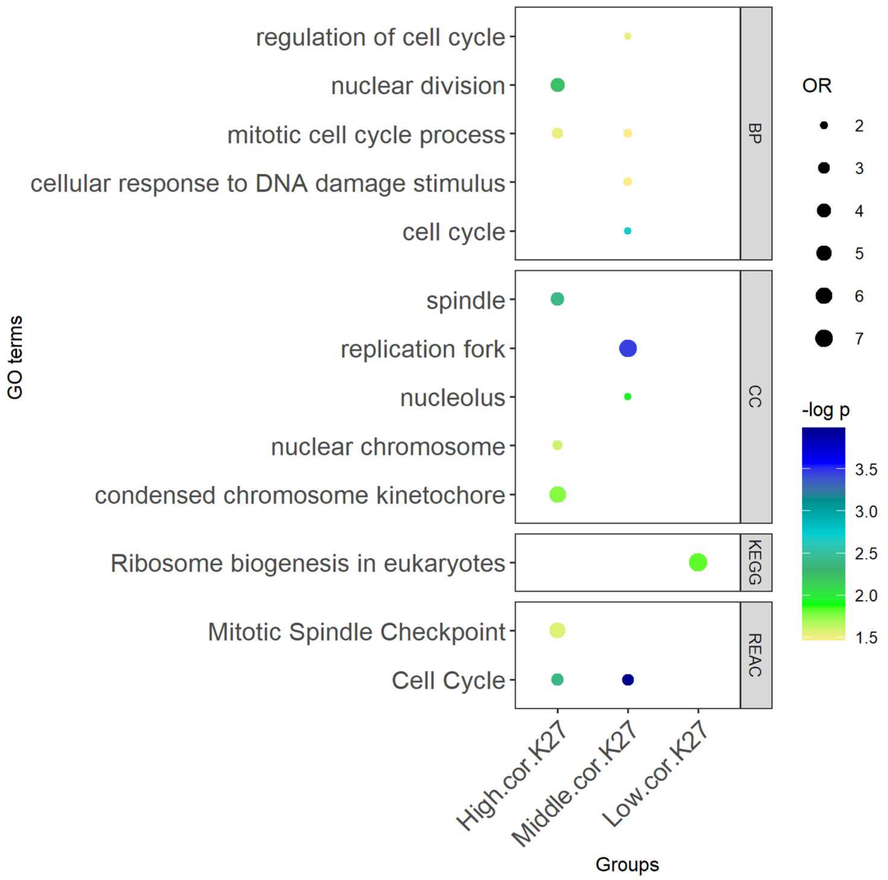

Supplementary Figure S12: Gene ontology (GO) analysis for cell cycle genes correlated with H3K27me3. The results show GO biological process (BP), cellular component (CC) terms, and KEGG and REACTOME pathways significantly enriched (*p*-values < 0.05) for cell cycle genes divided in three different groups; high correlation ($\rho > 0.2$), middle correlation ($\rho > -0.2$ and $\rho < 0.2$), and low correlation ($\rho < -0.2$). Correlation values are the results of Spearman's correlation analysis of RNA-seq expression against H3K27me3 signal.

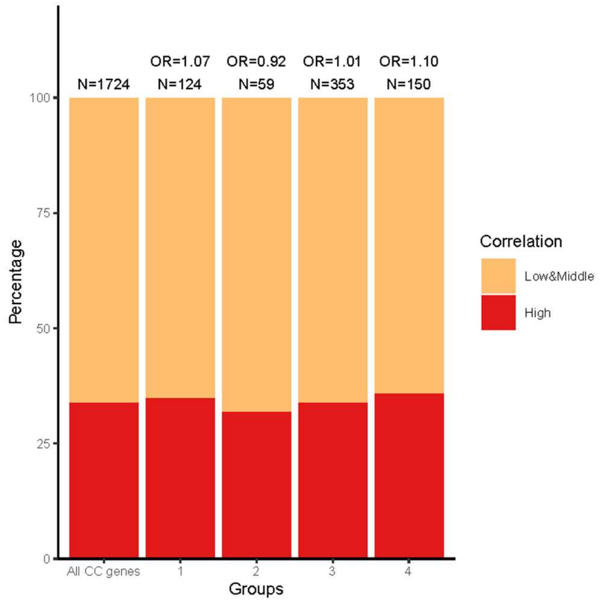

Supplementary Figure S13: Odds ratios from Fisher's exact tests for H3K4me3. We compared the fraction of highly correlated genes in the four gene groups (Figure 3D) with all cell cycle (CC) genes with H3K4me3 signals in their TSS (n = 1734). *P*-values (ns): group 1 p = 0.77, group 2 p = 0.89, group 3 p = 0.95, and group 4 p = 0.59.

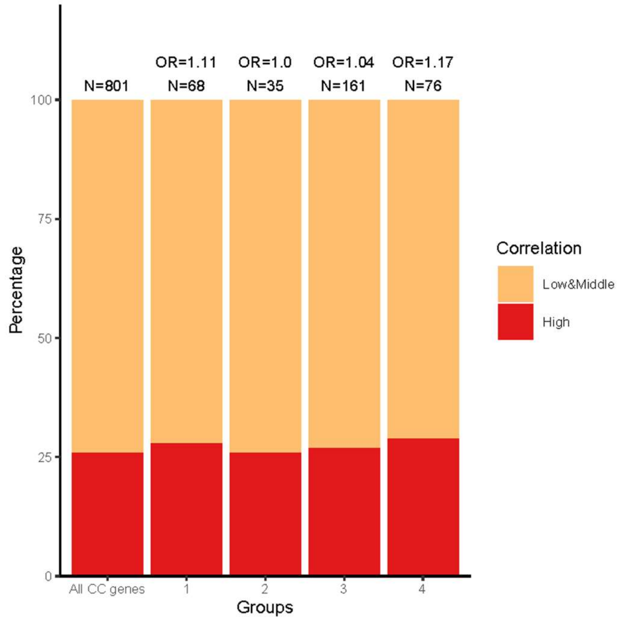

Supplementary Figure S14: Odds ratios from Fisher's exact tests for H3K27me3. We compared the fraction of highly correlated genes in the four gene groups (Figure 3D) with all cell cycle (CC) genes with H3K27me3 signals in their TSS (n = 801). *P*-values (ns): group 1 p = 0.77, group 2 p = 1, group 3 p = 0.84, and group 4 p = 0.58.

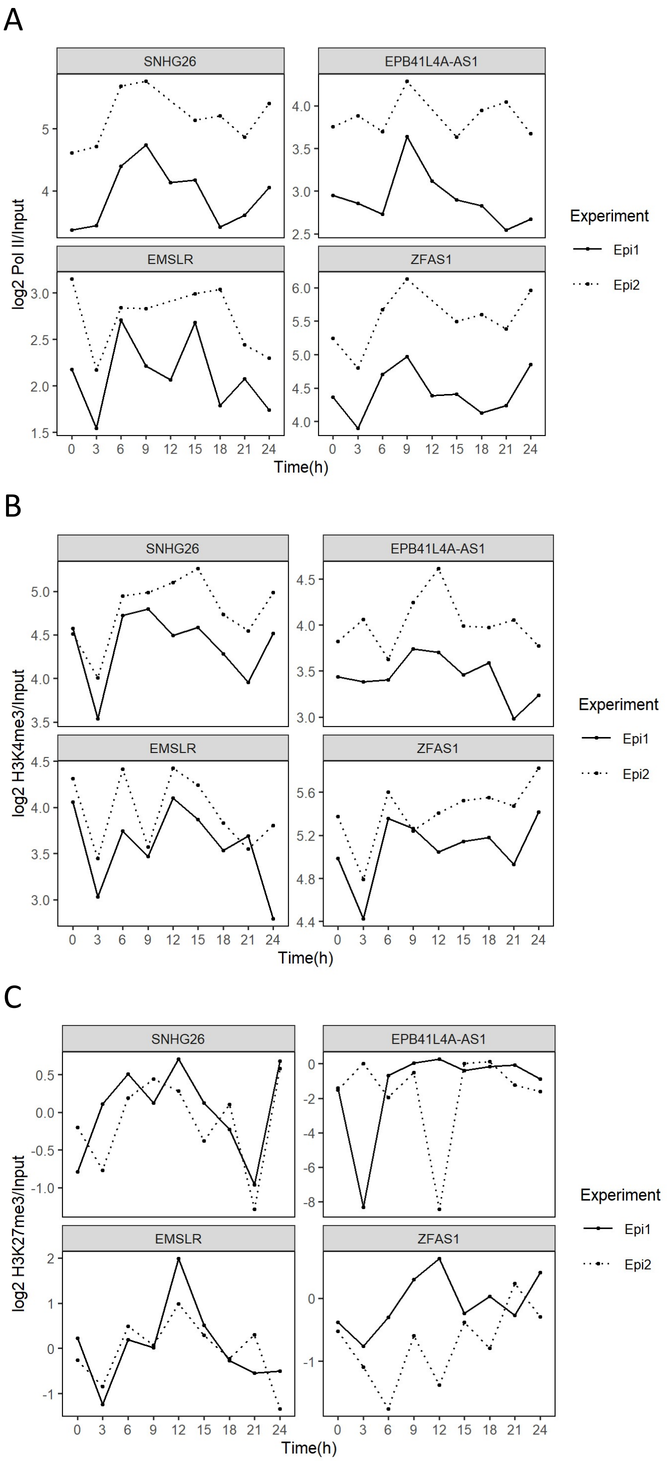

Supplementary Figure S15: ChIP-seq data for the four lncRNA candidates. (A) Pol II, (B) H3K4me3, and (C) H3K27me3 ChIP-seq profiles for *SNHG26*, *EPB41L4A-AS1*, *EMSLR*, and *ZFAS1*.

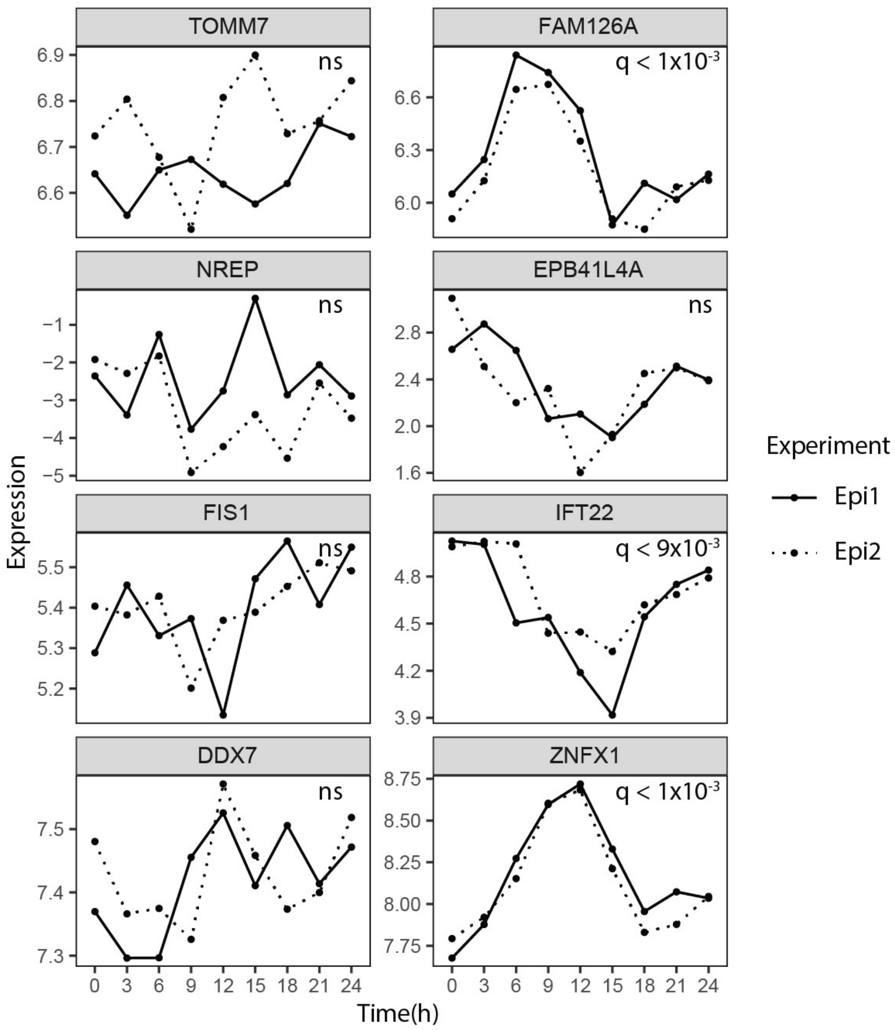

Supplementary Figure S16: RNA-seq profiles for lncRNA neighboring genes. *SNHG26* (*TOMM7* and *FAM126A*), *EPB41L4A-AS1* (*NREP* and *EPB41L4A*), *EMSLR* (*FIS1* and *IFT22*), and *ZFAS1* (*DDX7* and *ZNFX1*). q = the false discovery rate.

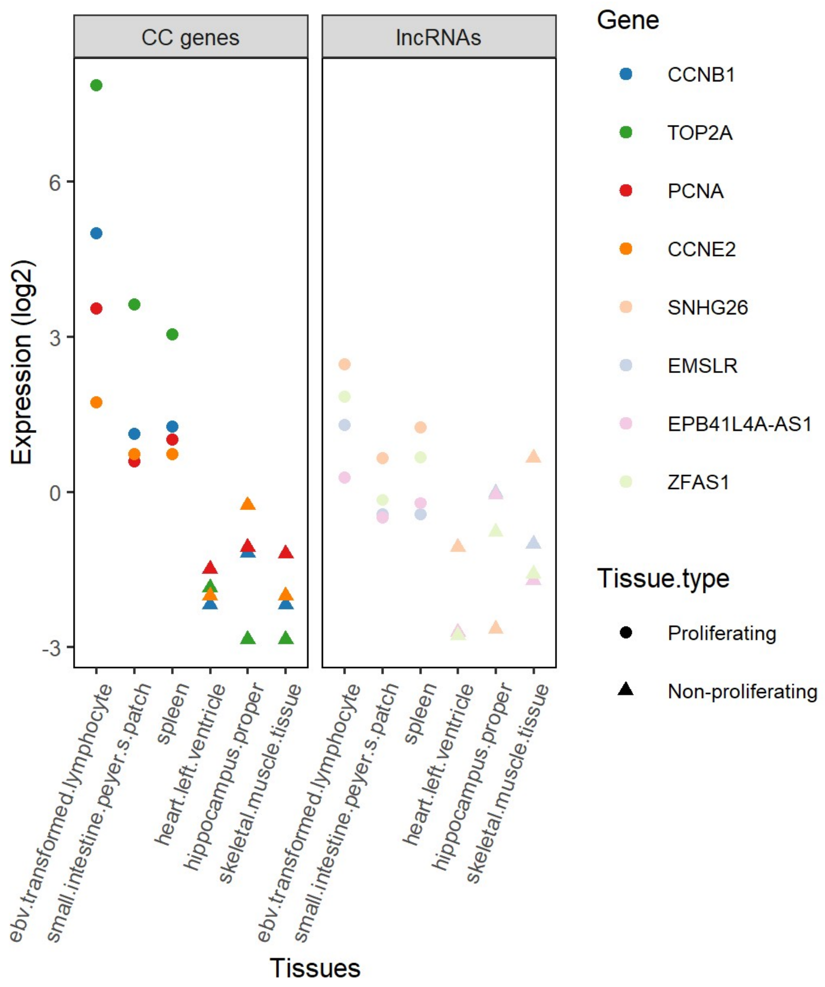

Supplementary Figure S17: Expression data for known cell cycle genes and the four lncRNA candidates in proliferating and non-proliferating tissues. Relative tissue expression (log transcript per kilobase million) of known cell cycle genes[1] and our cell cycle lncRNAs in selected tissues from the Genotype-Tissue Expression (GTEx) project. Gene expression values were normalized to relative expression values by subtracting the gene's average expression across all GTEx tissues.

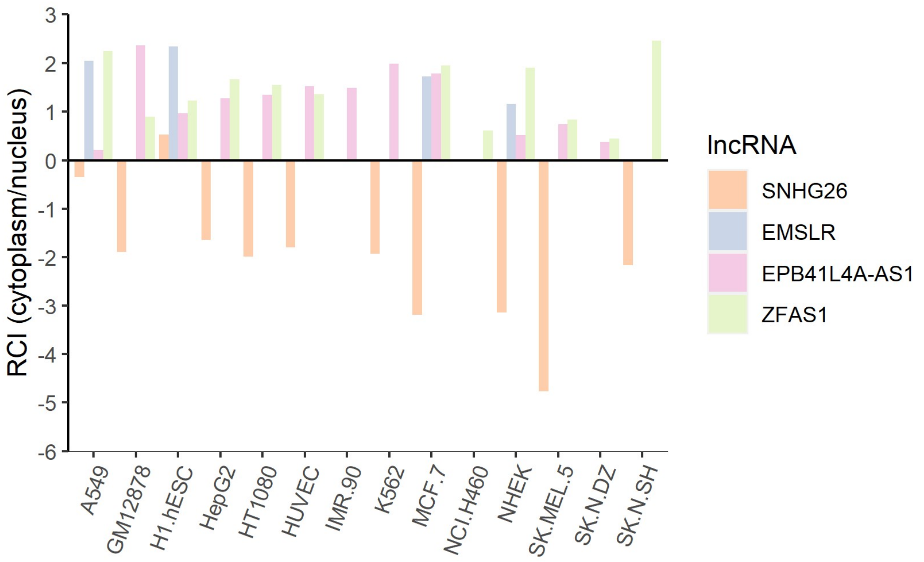

Supplementary Figure S18: Subcellular localization of lncRNA candidates. Data are from the lncAtlas (https://lncatlas.crg.eu/) and display the subcellular localization based on a relative concentration index (RCI) of RNA between cytoplasm and nucleus.

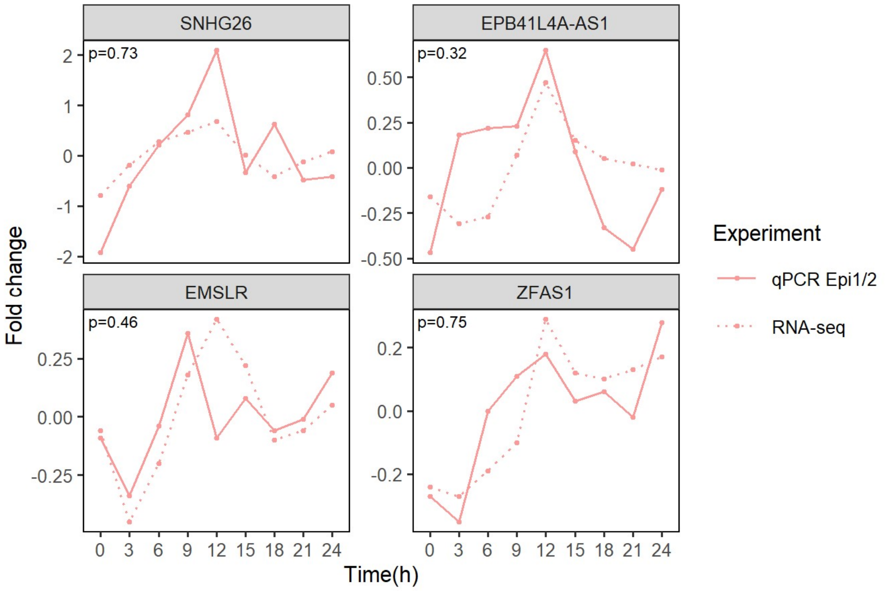

Supplementary Figure S19: Technical validation of RNA-seq expression for *SNHG26*, *EPB41L4A-AS1*, *EMSLR*, and *ZFAS1*. Spearman's correlation coefficients (ρ) were calculated based on the mean RNA-seq expression per time point and mean RT-qPCR fold change per time point from original experiments (Epi1, Epi2).

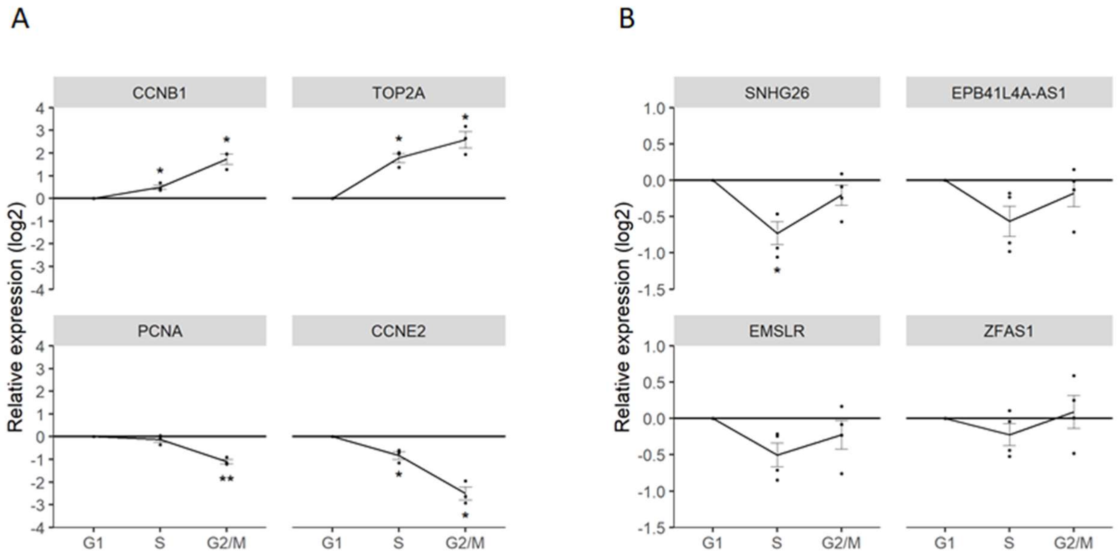

Supplementary Figure S20: Fluorescence-activated cell sorting (FACS) analysis of HaCaT cells. Relative expression (log2) for (A) *CCNB1*, *TOP2A*, *PCNA*, and *CCNE2* (n=3) and (B) *SNHG26*, *EPB41L4A-AS1, EMSLR*, and *ZFAS1* (n=4) as measured by RT-qPCR. Error bars represent standard error of mean (SEM). * $p \leq 0.05$; ** $p \leq 0.01$ (Welch's *t*-test).

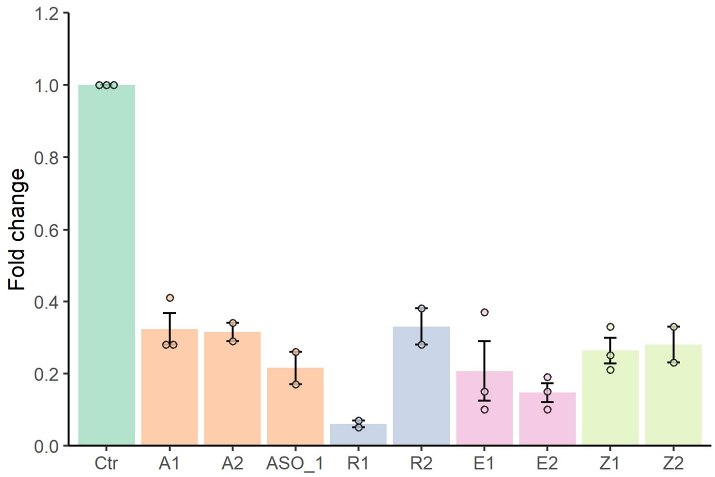

Supplementary Figure S21: Percentage downregulation of the lncRNA candidates. *SNHG26* (siRNAs; A1 and A2, and ASO_1), *EMSLR* (siRNAs; R1 and R2), *EPB41L4A-AS1* (siRNAs; E1 and E2), and *ZFAS1* (siRNAs; Z1 and Z2). Data are presented as fold change expressions of target lncRNAs following siRNA/ASO-treatment relative to control-treated cells as measured by RT-qPCR in HaCaT cells. Bars and error bars are mean and SEM of two or more independent replicates.

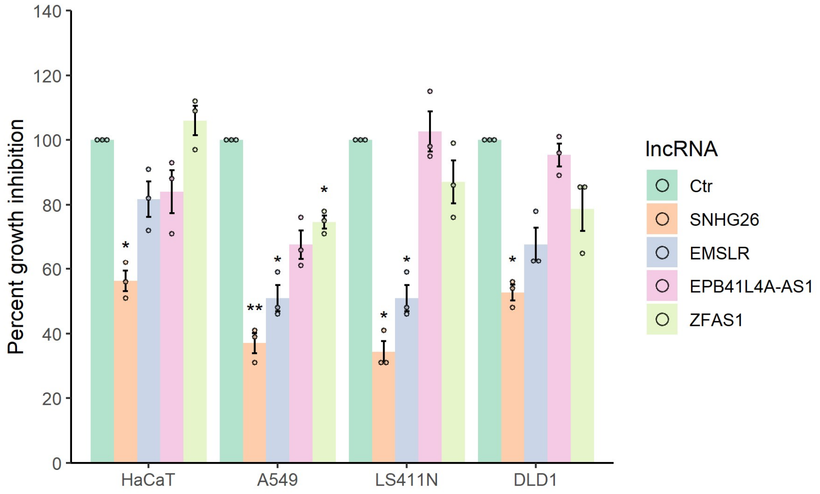

Supplementary Figure S22: Proliferation assay results. Effect of siRNA/ASO-mediated knockdown of *SNHG26* (ASO_1), *EMSLR* (siRNA R2), *EPB41L4A-AS1* (siRNA E2), and *ZFAS1* (siRNA Z2) on proliferation in four different cell lines. Data are the number of cells following siRNA/ASO-treatment relative to control-treated cells (percentage of control) as measured by cell counting. Bars and error bars are mean and SEM of three independent replicates. * $p \leq 0.05$; ** $p \leq 0.01$ (Welch's *t*-test, *p*-values were Bonferroni corrected for multiple testing). ANOVA *p*-values from the hierarchical, linear model: *SNHG26* p = 8.76-06, *EMSLR* p = 0.001, *EPB41L4A-AS1* p = 0.137, and *ZFAS1* p = 0.090.

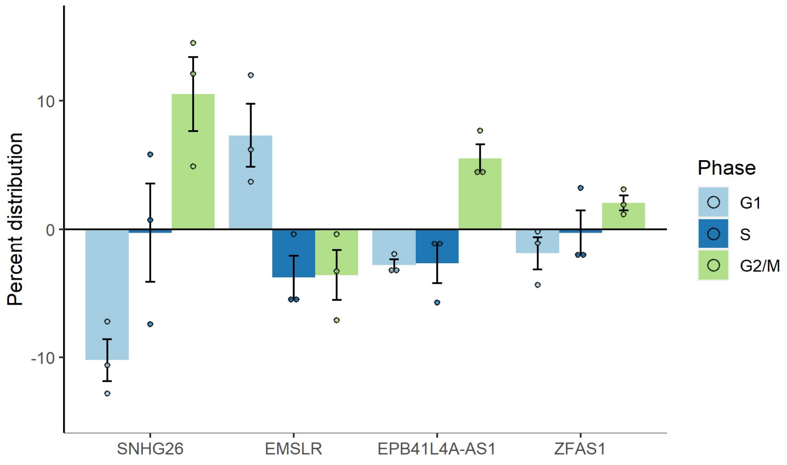

Supplementary Figure S23: Cell cycle phase distribution results. The distribution of cells in G1, S, and G2/M cell cycle phases in response to siRNA-mediated knockdown of *SNHG26* (siRNA A2), *EMSLR* (siRNA R2), *EPB41L4A-AS1* (siRNA E2), and *ZFAS1* (siRNA Z2) in HaCaT cells. Data are the difference in percentages of G1, S, and G2/M cells of siRNA-treated HaCaT cells to those of control-treated HaCaT cells. Bars and error bars are mean and SEM of three independent replicates. Welch's *t*-test *p*-values (non-significant) were Bonferroni corrected for multiple testing: *SNHG26* G2/M: 0.271, *EPB41L4A-AS1* G1: 0.095, and *EPB41L4A-AS1* G2/M: 0.143. ANOVA *p*-values were calculated from a hierarchical, linear model assuming a random effect for two independent siRNAs: *SNHG26*: G1: 3.08e-06, S: 0.669, G2/M: 1.95e-06, *EMSLR*: G1: 0.002, S: 0.006, G2/M: 0.629, *EPB41L4A-AS1* G1: 6.01e-05, S: 5.81e-02, G2/M: 2.30e-06, *ZFAS1*: G1: 0.014, S: 0.65, G2/M: 0.006.

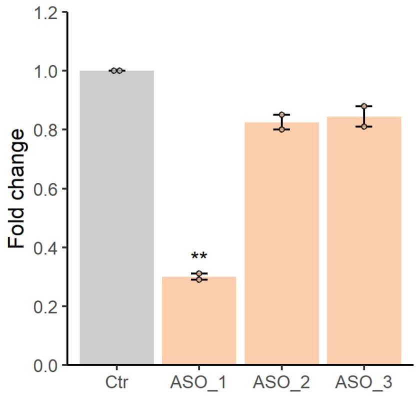

Supplementary Figure S24: Percentage downregulation of *SNHG26* by using three ASOs with different target sequences. Data are presented as fold change expressions of *SNHG26* following ASO treatment relative to control-treated cells as measured by RT-qPCR in HaCaT. Bars and error bars are mean and SEM of two independent replicates. ** $p \leq 0.01$ (Welch's *t*-test).

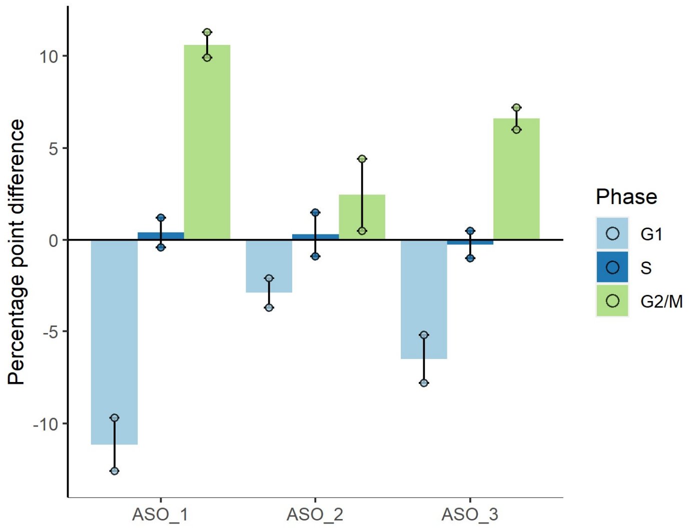

Supplementary Figure S25: Cell cycle phase distribution in G1, S, and G2/M cell cycle phases after ASO-mediated knockdown of *SNHG26* in HaCaT cells. Data are the difference in percentages of G1, S, and G2/M cells of ASO-treated HaCaT cells to those of control-treated HaCaT cells. Bars and error bars are mean and SEM of two independent replicates. Individual *p*-values were non-significant (Welch's *t*-test, Bonferroni corrected for multiple testing). ANOVA *p*-values across the three ASOs with different target sequences: G1: 1.84e-04, S: 0.91, G2/M: 2.75e-04.

Supplementary tables

Supplementary Table S1: Primer sequences of selected genes for Pol II ChIP-qPCR validations studies. S = sense, AS = antisense

Gene	Sequence	PCR product (bp)
PCNA	S-5'-TAGCTGGTTTCGGCTTCAGG-3'	106
	AS-5'-TAAACGGTTGCAGGCGTAG-3'	
TOP2A	S-5'-AAGCGACTAAACAGGCAGG-3'	103
	AS-5'-GGCTAAAGGAAGGTTCAAGTG-3'	
CCNB1	S-5'-ACGAACAGGCCAATAAGGAG-3'	103
	AS-5'-ACCCAGCAGAAACCAACAG-3'	
CCNE2	S-5'-GCTTTCTTTCCTCCCACATC-3'	102

Supplementary Table S2: Long non-coding RNAs with RNA-seq/ChIP-seq Spearman's correlation values. Gene type is from the Human Genes GRCh38.p13 database.

EnsemblGene	EntrezGene	Phase	Gene type	Cor PolII	Cor H3K4me3	Cor H3K27me3
ENSG00000228649	SNHG26	G2/M	lncRNA	0.84	0.49	0.70
ENSG00000255121	RP11-110I1.12	S	lncRNA	0.78	0.44	0.09
ENSG00000163597	SNHG16	M/G1	lncRNA	0.76	0.68	NA
ENSG00000259230	CTD-2555C10.3	S	lncRNA	0.68	0.64	NA
ENSG00000173209	AHSA2	S	pseudogene	0.67	0.26	NA
ENSG00000275481	RP11-474P2.6	S	lncRNA	0.66	0.55	NA
ENSG00000230133	FLJ33581	G2	lncRNA	0.66	NA	0.27
ENSG00000257167	TMPO-AS1	S	lncRNA	0.64	-0.03	NA
ENSG00000251669	FAM86EP	G1/S	pseudogene	0.64	0.46	0.10
ENSG00000182796	TMEM198B	S	pseudogene	0.62	0.13	0.12
ENSG00000263753	LINC00667	S	lncRNA	0.58	0.35	0.62
ENSG00000256268	RP11-221N13.3	S	lncRNA	0.55	-0.26	NA
ENSG00000238105	GOLGA2P5	S	pseudogene	0.55	NA	NA
ENSG00000204934	ATP6V0E2-AS1	S	lncRNA	0.54	0.55	0.51
ENSG00000235748	SEPT14P12	G2	pseudogene	0.53	0.19	NA
ENSG00000250685	RP11-486L19.2	S	lncRNA	0.53	0.14	-0.20
ENSG00000237870	AC073130.1	S	lncRNA	0.51	0.15	NA
ENSG00000240541	TM4SF1-AS1	S	lncRNA	0.47	-0.01	NA
ENSG00000213742	ZNF337-AS1	S	lncRNA	0.47	0.20	NA
ENSG00000245213	RP11-10K16.1	S	lncRNA	0.46	0.26	0.20
ENSG00000250899	RP11-253E3.3	G1/S	lncRNA	0.45	0.01	-0.11
ENSG00000281398	SNHG4	M/G1	lncRNA	0.42	0.42	NA
ENSG00000214049	UCA1	M/G1	lncRNA	0.42	0.05	-0.13
ENSG00000204177	BMS1P1	S	pseudogene	0.40	0.00	NA
ENSG00000226312	CFLAR-AS1	S	lncRNA	0.38	0.04	NA
ENSG00000235897	TM4SF19-AS1	S	lncRNA	0.38	0.49	NA
ENSG00000270332	SMC2-AS1	G1/S	lncRNA	0.38	0.44	0.23
ENSG00000197182	MIRLET7BHG	S	lncRNA	0.37	0.21	NA

ENSG00000278864	RP11-45M22.2	M/G1	TEC	0.37	0.25	-0.27
ENSG00000248092	NNT-AS1	G1/S	lncRNA	0.37	0.17	NA
ENSG00000267100	ILF3-AS1	G1/S	lncRNA	0.37	0.33	NA
ENSG00000204054	LINC00963	S	lncRNA	0.35	0.18	NA
ENSG00000264538	SUZ12P1	G1/S	pseudogene	0.35	0.35	NA
ENSG00000241769	LINC00893	S	lncRNA	0.34	0.01	-0.59
ENSG00000225138	CTD-2228K2.7	S	lncRNA	0.29	0.00	-0.06
ENSG00000280239	CTB-50L17.8	G1/S	TEC	0.28	0.37	NA
ENSG00000214826	DDX12P	S	pseudogene	0.28	0.13	NA
ENSG00000255198	SNHG9	G1/S	lncRNA	0.27	0.43	-0.09
ENSG00000225733	FGD5-AS1	S	lncRNA	0.26	-0.01	NA
ENSG00000230479	AP000695.6	S	lncRNA	0.25	0.17	NA
ENSG00000233016	SNHG7	M/G1	lncRNA	0.25	0.29	0.27
ENSG00000237886	NALT1	S	lncRNA	0.25	0.17	NA
ENSG00000232445	EMSLR	M/G1	lncRNA	0.25	0.33	0.53
ENSG00000274605	RP11-12G12.7	S	lncRNA	0.24	-0.18	0.05
ENSG00000268573	RP11-158H5.7	S	lncRNA	0.24	-0.30	NA
ENSG00000274173	RP4-568C11.4	S	lncRNA	0.21	0.06	0.45
ENSG00000254635	WAC-AS1	S	lncRNA	0.20	-0.15	NA
ENSG00000197989	SNHG12	M/G1	lncRNA	0.20	0.13	0.44
ENSG00000215417	MIR17HG	M/G1	lncRNA	0.19	0.12	-0.40
ENSG00000177410	ZFAS1	G1/S	lncRNA	0.18	0.36	0.43
ENSG00000224032	EPB41L4A-AS1	M/G1	lncRNA	0.16	0.51	0.31
ENSG00000233901	LINC01503	S	lncRNA	0.16	0.29	-0.17
ENSG00000269893	SNHG8	G1/S	lncRNA	0.15	0.24	0.38
ENSG00000255717	SNHG1	G1/S	lncRNA	0.14	-0.31	0.06
ENSG00000267080	ASB16-AS1	S	lncRNA	0.13	0.04	NA
ENSG00000259488	RP11-154J22.1	G1/S	lncRNA	0.12	-0.02	-0.31
ENSG00000154874	CCDC144B	S	pseudogene	0.11	-0.28	0.16
ENSG00000226950	DANCR	G1/S	lncRNA	0.11	-0.09	0.07
ENSG00000251562	MALAT1	S	lncRNA	0.10	0.28	NA
ENSG00000254531	FLJ20021	S	lncRNA	0.08	-0.27	NA
ENSG00000272468	RP1-86C11.7	M/G1	lncRNA	0.08	-0.22	NA
ENSG00000236384	LINC00479	S	lncRNA	0.07	-0.05	NA
ENSG00000254837	AP001372.2	S	lncRNA	0.06	-0.28	0.00
ENSG00000260260	SNHG19	M/G1	lncRNA	0.06	0.28	-0.01
ENSG00000234327	AC012146.7	S	lncRNA	0.05	-0.21	0.25
ENSG00000231298	LINC00704	S	lncRNA	0.04	0.08	NA
ENSG00000268798	CTB-25B13.5	M/G1	lncRNA	0.04	-0.03	0.18
ENSG00000268364	SMC5-AS1	S	lncRNA	0.04	-0.45	NA
ENSG00000261578	RP11-21L23.2	G2/M	lncRNA	0.04	NA	NA
ENSG00000204860	FAM201A	S	lncRNA	0.03	-0.34	NA
ENSG00000180385	EMC3-AS1	G1/S	pseudogene	0.00	-0.10	-0.18
ENSG00000241015	TPM3P9	G1/S	pseudogene	0.00	0.52	0.12
ENSG00000236751	LINC01186	S	lncRNA	0.00	-0.18	-0.50
ENSG00000251381	LINC00958	G1/S	lncRNA	-0.02	0.23	0.28

ENSG00000234741	GAS5	M/G1	lncRNA	-0.02	0.08	-0.49
ENSG00000258727	RP11-66N24.3	S	lncRNA	-0.05	-0.30	-0.07
ENSG00000279873	LINC01126	S	lncRNA	-0.07	-0.02	NA
ENSG00000230937	MIR205HG	S	lncRNA	-0.07	0.17	0.18
ENSG00000203709	C1orf132	S	lncRNA	-0.08	-0.44	-0.36
ENSG00000249042	CTD-2015H6.3	S	lncRNA	-0.12	-0.04	NA
ENSG00000253669	KB-1732A1.1	S	lncRNA	-0.13	-0.30	NA
ENSG00000237813	AC002066.1	S	lncRNA	-0.15	-0.52	NA
ENSG00000232995	RGS5	S	lncRNA	-0.17	-0.20	NA
ENSG00000187951	ARHGAP11B	G2	lncRNA	-0.18	-0.29	NA
ENSG00000206337	HCP5	G2	lncRNA	-0.21	-0.42	NA
ENSG00000235609	AF127936.9	G1/S	lncRNA	-0.23	NA	NA
ENSG00000260708	CTA-29F11.1	M/G1	lncRNA	-0.28	-0.19	NA
ENSG00000260032	LINC00657	S	lncRNA	-0.29	-0.39	NA
ENSG00000263412	RP5-890E16.2	G1/S	lncRNA	-0.32	-0.01	NA
ENSG00000269896	RP4-740C4.5	S	pseudogene	-0.35	0.27	0.00
ENSG00000227036	LINC00511	S	lncRNA	-0.38	-0.37	-0.16
ENSG00000230844	ZNF674-AS1	M/G1	lncRNA	-0.42	-0.52	-0.51
ENSG00000203875	SNHG5	G1/S	lncRNA	-0.61	-0.35	NA
ENSG00000244879	GABPB1-AS1	S	lncRNA	-0.80	-0.78	NA
ENSG00000204789	ZNF204P	S	pseudogene	NA	NA	0.21
ENSG00000231007	CDC20P1	M/G1	pseudogene	NA	NA	0.18
ENSG00000234176	HSPA8P1	M/G1	pseudogene	NA	NA	-0.23

TEC = To be Experimentally confirmed

Supplementary Table S3: Info about the four lncRNA candidates.

Ensembl Gene ID	lncRNA names	Localization	Strand	Transcripts	Coding probability (CP*)
ENSG00000224032	EPB41L4A-AS1<br>TIGA1	Chr5: 112,160,526-112,164,818	Plus	5	0.1416
ENSG00000177410	ZFAS1	Chr20: 49,278,178-49,299,600	Plus	14	0.0730
ENSG00000232445	EMSLR<br>RP11-132A1.4<br>AC006329.1	Chr7: 101,308,270-101,314,800	Plus	2	0.0167
ENSG00000228649	SNHG26<br>AC005602.5	Chr7: 22,854,126-22,872,945	Plus	13	0.0077

*CP values are results from the Coding Potential Assessment Tool. Human CP cutoff is 0.364. CP >= 0.364 indicates coding sequence, whereas CP < 0.364 indicates noncoding sequence[2].

Supplementary Table S4: Primers used for RT-qPCR. Qiagen QuantiTect primer assays (249900) were used for mRNA expression analysis and Qiagen RT^2 lncRNA PCR assays (330701) were used for lncRNA expression analysis.

Gene	Primer assay	Cat.no
GAPDH	Hs_GAPDH_1_SG QuantiTect Primer Assay	QT00079247
PCNA	Hs_PCNA_1_SG QuantiTect Primer Assay	QT00024633
TOP2A	Hs_TOP2A_1_SG QuantiTect Primer Assay	QT00037632
CCNB1	Hs_CCNB1_1_SG QuantiTect Primer Assay	QT00006615
CCNE2	Hs_CCNE2_1_SG QuantiTect Primer Assay	QT00063511
SNHG26	RT^2 lncRNA qPCR Assay for Human LOC101927841	LPH07369A-200
EPB41L4A-AS1	RT^2 lncRNA qPCR Assay for Human EPB41L4A-AS1	LPH04387A-200
EMSLR	RT^2 lncRNA qPCR Assay for Human EMSLR	LPH09823A-200
ZFAS1	RT^2 lncRNA qPCR Assay for Human ZFAS1	LPH01402A-200

Supplementary Table S5: Human control qPCR primer sets from Active Motif. Human control qPCR primer sets are designed to serve as positive or negative ChIP controls when performing ChIP with human samples.

Primer set	Location	Cat.no	Control for
Human positive control primer set ACTB-2	ACTB promoter	71005	H3K4me3 and Pol II
Human positive control primer set GAPDH-2	GAPDH intron 1	71006	H3K4me3 and Pol II
Human positive control primer set MYT1	MYT1 promoter	71007	H3K27me3
Human positive control primer set CCND2	CCND2 promoter	71008	H3K27me3
Human negative control primer set 1	Gene desert on chr. 12	71001	H3K4me3, Pol II and H3K27me3

Supplementary Table S6: Antisense oligos and siRNAs used in RNA interference experiments.

Target lncRNA	Alias	Cat.no	Producer	Sense sequence	Target
EPB41L4A-AS1	E1	CTM-431273	Dharmacon	ACAGUGAGGAUGUGAAUAAUU	
EPB41L4A-AS1	E2	CTM-431274	Dharmacon	GUGAGGAUGUGAAUAAAUAUU	
ZFAS1	Z1	Custom	Sigma	CUUUGAUUGAACCAGGAUG	
ZFAS1	Z2	Custom	Sigma	AGUAGAAUAUAUAUAUACA	
EMSLR	R1	Custom	Sigma	CACAGGAUGUGCAGAUCUC	
EMSLR	R2	Custom	Sigma	GAAAUGUCAUAUAGAGAAU	
SNHG26	A1	Custom	Sigma	CUGCAUUCCUUGUCCUCAU	
SNHG26	A2	CTM-431277	Dharmacon	GAAAGGAGGCUGAGUAAUUUU	
SNHG26	ASO_1	339517	Qiagen	GGAAGCCACAGTAGTA	Exon 4
SNHG26	ASO_2	339517	Qiagen	CCTGATTAAACGTTGG	Intron 1
SNHG26	ASO_3	339517	Qiagen	CACTGCGGAGATCAAG	Exon 1
siRNA neg. control	Ctr	SIC001	Sigma		
ASO neg. control A	Ctr	339515	Qiagen	AACACGTCTATACGC	

References

1 Whitfield, M. L. *et al.* Identification of genes periodically expressed in the human cell cycle and their expression in tumors. *Mol Biol Cell* **13**, 1977-2000, doi:10.1091/mbc.02-02-0030 (2002).

2 Chakraborty, S., Deb, A., Maji, R. K., Saha, S. & Ghosh, Z. LncRBase: an enriched resource for lncRNA information. *PloS one* **9**, e108010, doi:10.1371/journal.pone.0108010 (2014).
